# Factors impacting on progress towards elimination of transmission of schistosomiasis japonica in China

**DOI:** 10.1186/1756-3305-5-275

**Published:** 2012-12-03

**Authors:** Yi-Biao Zhou, Song Liang, Qing-Wu Jiang

**Affiliations:** 1 Department of Epidemiology, School of Public Health, Fudan University, 138 Yi Xue Yuan Road, Shanghai 200032, China; 2 Key Laboratory of Public Health Safety, Ministry of Education (Fudan University), 138 Yi Xue Yuan Road, Shanghai 200032, China; 3 Department of Environmental and Global Health, College of Public Health and Health Professions, University of Florida, Gainesville, FL, 32610, USA; 4 Emerging Pathogens Institute, University of Florida, Gainesville, FL, 32610, USA

**Keywords:** *Schistosoma japonicum*, Integrated control strategy, Transmission interruption

## Abstract

Over the past decades China has made a great stride in controlling schistosomiasis, eliminating transmission of *Schistosoma japonicum* in 5 provinces and remarkably reducing transmission intensities in the rest of the seven endemic provinces. Recently, an integrated control strategy, which focuses on interventions on humans and bovines, has been implemented throughout endemic areas in China. This strategy assumes that a reduction in transmission of *S. japonicum* from humans and bovines to the intermediate *Oncomelania* snail host would eventually block the transmission of this parasite, and has yielded effective results in some endemic areas. Yet the transmission of *S. japonicum* is relatively complicated – in addition to humans and bovines, more than 40 species of mammalians can serve as potential zoonotic reservoirs. Here, we caution that some factors – potential roles of other mammalian reservoirs and human movement in sustaining the transmission, low sensitivity/specificity of current diagnostic tools for infections, praziquantel treatment failures, changes in environmental and socio-economic factors such as flooding in key endemic areas - may pose great obstacles towards transmission interruption of the parasite. Assessing potential roles of these factors in the transmission and implications for current control strategies aiming at transmission interruption is needed.

## Review

### Background

Worldwide, *Schistosoma* infections remain a serious public health problem, infecting more than 200 million people in approximately 76 countries with a loss of 1·53 million disability-adjusted life years (DALYs) [1,2]. Of the species of schistosomes infecting humans, *Schistosoma japonicum,* distributed in southern China, the Philippines, and parts of Indonesia, is the only schistosome species whose transmission is zoonotic [1]. China’s schistosomiasis control program was initiated in the mid-1950s, and has undergone three stages [3-8]. The first stage (from the mid-1950s to mid-1980s) focused on transmission control, and the control program included chemotherapy, snail control primarily by environmental management or the use of molluscicide, improving access to clean water and sanitary facilities [3,5]. By the end of the 1980s, schistosomiasis transmission was eliminated in four endemic provinces but the prevalence of disease remained high in the rest of eight endemic provinces [3,5]. The second stage (from the mid-1980s to the early 2000s) focused on morbidity control, and large-scale chemotherapy (due to the introduction of praziquantel) served as the backbone of The National Schistosomiasis Control Program, which was complemented with health education and continued snail control [3-8]. In the mid-1990s, schistosomiasis transmission was eliminated in one of the remaining eight endemic provinces [3]. Despite continued and intensified control efforts, controlling transmission of the disease in the remaining seven endemic provinces (Hubei, Hunan, Anhui, Jiangxi, Jiangsu Sichuan and Yunnan provinces) has been challenging, and, eliminating the transmission in these regions seems a daunting task [3]. A number of studies reported that bovines are the primary infection source of *S. japonicum* transmission in China [3,9-12]. Based on this, the third stage of control, also called integrated control, aiming at reducing the roles of bovines and humans as infection sources, has been implemented in China since 2005. With renewed emphasis on transmission control and ultimately transmission interruption, control programs at this stage consist of agricultural mechanization, fencing bovines (particularly water buffalos), chemotherapy on both humans and bovines, health education, provision of clean water and improved sanitation [3]. This national strategy has so far achieved promising results and two notable patterns have been observed - in some endemic areas the prevalence of *S. japonicum* infection in humans was brought below 1% [3,13], whereas in other endemic regions with implementation of the same strategy, human infections were sustained at around 3% [3,14]. More recently, there was a discussion on the generalizability of this national integrated strategy to control and eventually interrupt the transmission of *S. japonicum*, which was based on the studies in lake regions of China, and in hilly and mountainous regions of Sichuan and Yunnan the transmission ecology is quite different [15]. Concern has been raised that, in addition to humans and bovines, there might be other factors that implicate the transmission but are neglected [14,16,17]. Here we summarize these factors based on a systematic review of published literature and focus on non-bovine animal hosts, human movements, low sensitivity/specificity of current diagnostic tools for schistosome infection, and praziquantel treatment failure and their implications for the current schistosomiasis control strategy in China. The following databases were used for identifying relevant articles: PubMed, Cochrane Library, Science Citation Index Expanded, China National Knowledge Infrastructure, Wanfang Database, VIP Database, and ProQuest. No restriction was applied to year of publication. All titles and abstracts were independently examined and screened by two reviewers to check their eligibility for inclusion in the review. An article was included in the tables of this paper if it met one of the following criteria included: (1) report on prevalence of infection of *S. japonicum* in non-bovine animal hosts in China; (2) report on prevalence of infection of *S. japonicum* in migrant populations in China. Upon inclusion, full papers were retrieved and reviewed by the same two reviewers.

### Non-bovine animal hosts

*S. japonicum*, unlike *S. mansoni* and *S. haematobium*, is widely recognized as a zoonotic parasite. Over 40 species of wild and domesticated animals can serve as reservoir hosts for this parasite [9]. The zoonotic nature of *S. japonicum* complicates control efforts for the parasite. In China a number of non-human mammalian hosts, such as bovines, pigs, dogs, cats, goats/sheep, and rats, can serve as reservoir hosts and are of potential public health importance (Table 1) [10,11,16,18-26]. In addition to bovines, relatively high infection levels were also found in other mammalian hosts in some endemic areas (Table 1) [10,11,16,18-26]. High prevalences of *S. japonicum* infections in rats (59.8%) and in *Oncomelania* snails (0.4-3.1%) were reported in two islands located in the Yangtze River [19]. The two islands have only emerged in the last 40 years, humans do not inhabit these islands. High infections together with estimates of high transmission index (measured by excreted eggs per day) were also reported in dogs, cats, and rats in a hilly area of China where no infected bovines were found [16]. A similar pattern was also reported in Samar Province, the Philippines, where high prevalences and intensities of *S. japonicum* infection were also observed in dogs (14.9% prevalence) and rats (29.5% prevalence), suggesting that dogs could potentially be a very important reservoir host of *S. japonicum* in that region [27,28]. A modeling study indicated that rats may also play a role in human infections in Samar [27]. These findings suggested that the transmission of *S. japonicum* might occur in the absence of bovines. In addition, high genetic variation in *S. japonicum* was found in the marshland and lake endemic regions of China (where bovines are considered as an important source of infection) and two main genetic clusters were discovered, separating the strain in bovines and humans from that in goats, dogs, pigs and cats [18]. The result indicated that *S. japonicum* from bovines and humans might be different from that from other mammalian animals in lake and marshland areas of southern China. Also notable was that the prevalence of *S. japonicum* infection in pigs, dogs and cats increased when the bovines with high prevalence of infection were removed from the endemic area [16]. The strain from bovines and humans may be more prevalent than those from other mammalian animals in lake and marshland areas of southern China. When the predominant strain is completely or partly removed from endemic areas, the other strain may become predominant. Further studies are needed to address this question, for example, molecular epidemiological research should be performed to address whether the strains cross-infect humans and evaluate relative contribution of different animal reservoirs to sustain the transmission. Without improved knowledge on these issues, the reliance of human- and bovine-oriented control to achieve full control of infection sources might miss some other important sources of infection.

**Table 1:** **Reported prevalence of infection of *Schistosoma japonicum *among different non-human hosts in China**

**Author and date of publication**	**Environmental type**	**Definitive hosts**									
		**Bovine**	**Pig**	**Dog**	**Goat/sheep**	**Cat**	**Donkey**	**Horse**	**Mule**	**Rodent**	**Rabbit**
Lu DB *et al*. (2010) [16]	Marshland*	35·5	0·0	4·8	55·0	0·0	-	-	-	0·0	-
	Marshland†	-	3·9	8·4	-	37·5	-	-	-	-	-
	Hill*	0·0	0·0	18·9	-	2·6	-	-	-	26·5	-
	Hill†	-	0·0	21·1	-	5·3	-	-	-	17·7	-
Wang TP *et al*. (2006) [18]	Marshland‡	26·9	7·4	2·7	33·3	0·0	-	-	-	-	-
	Marshland§	15·4	0·0	4·1	0·0	1·5	-	-	-	-	-
Xu GY *et al*. (1999) [19]	Marshland	-	-	-	-	-	-	-		59·8	-
Wang TP *et al*. (1997) [20]	Marshland	48·7	21·3	0·7	13·0	0·0	-	-	-	8·3	18·2
Sun LP *et al*. (1997) [21]	Marshland	22·1	26·7	-	-	-	-	-	-	-	-
Xu FS *et al*. (1995) [22]	Mountain	36·9	-	3·0	-	-	-			-	-
Su ZW *et al*. (1994) [23]	Lake	35·7	60·0	75·0	-	-	-	-	-	-	-
Gu XG *et al*. (1993) [24]	Mountain	36·9	-	3·0	-	-	-	-	-	0·9	-
Dai ZJ *et al*. (1991) [10]	Mountain	42·3	6·7	7·5	8·5	-	-	13·5	-	-	-
Zheng J *et al*. (1990) [11]	Mountain	17·3	13·2	8·2	4·2	0·0	4·3	3·4	3·2	1·4	-
Chen DJ *et al*. (1989) [25]	Mountain	15·2	17·1	75·0	10·8	-	-	4·7	-	57·1	-
Yao BY *et al*. (1989) [26]	Mountain	16·5	9·3	9·6	0·0	0·0	1·8	1·4	3·5	1·7	-

Note: * = 2006; †=2007; ‡= Chenqiao village; § = Guanghui village.

### Human movement

The second potential obstacle in moving towards the elimination of schistosomiasis transmission relates to human movement. Along with China’s rapid economic development and urbanization over the past decades, the country has witnessed the largest human movement, in particular rural–urban migration in the human history (Table 2) [29-37]. For example, the movement population of rural migrants moving to coastal cities is approximately 120 million [38]. Meanwhile, large population movements are also occurring between cities, in particular from small cities to big cities [38]. It has long been recognized that human movement plays an important role in the epidemiology of many infectious diseases [38-40]. For example, some infectious diseases (e.g. leishmaniasis, Chagas disease, lymphatic filariasis, and schistosomiasis) have emerged or re-emerged in urban areas or previously controlled regions, which have been linked to human movements [38,41].

**Table 2:** **Prevalence of infection of *Schistosoma japonicum *in different migrant populations**

**Author and date of publication**	**Movement type**	**Proportion of migration (%)**	**Prevalence (%)**
Chen GX *et al*. (2011) [29]	Migration from endemic areas to other areas	22·4-38·5	2·6-5·3
Wang ZC *et al*. (2008) [30]	Migration from non-endemic areas to endemic areas	>5·3	12·5
He JC *et al*. (2008) [31]	Migration from endemic areas to other areas	-	3·2
	Migration from non-endemic areas to endemic areas	-	3·0
Zhang YQ *et al*. (2003) [32]	Migration from endemic areas to other areas	29·9	26·0
Li YS *et al*. (2003) [33]	Fishing population	-	68·9
Chen GX *et al*. (2001) [34]	Migration from endemic areas to other areas	19·7	15·6
Zhang SQ *et al*. (1998) [35]	Migration from endemic areas to other areas	19·8-32·4	2·1-13·9
Zheng J *et al*. (1999) [36]	Fishing population	-	18·2-84·2
	Boatman	-	54·2-69·4
	Herdsman	-	41·2
Ross AG *et al*. (1997) [37]	Fishing population	-	22·4

The occurrence of schistosome transmission in urban areas or re-emergence of the transmission in previously controlled areas were most probably through infected migrants and endemic foci was present in large cities such as in Bamako, Kampala, Changsha and Dar el Salam [38,41]. The impact of human migration on the transmission of schistosomiasis has been reported. For example, a report in Brazil indicated that migrants play an important role in the transmission of schistosomiasis, and the presence of mobile populations (e.g. migrants, returnees and tourists) in endemic regions can also impact significantly on the control effectiveness of schistosomiasis due to insufficient health services [42]. Yet, this potential remains largely unknown in China. In addition to lack of information on the spatial and temporal patterns of population movements, little is available on the epidemiological impact of the movement patterns on transmission and maintenance of schistosomiasis in China. Hence, studies to understand how human movements (e.g. between endemic and endemic areas, endemic and non-endemic areas) impact the transmission and control are needed. In addition to human movement, livestock movement (e.g. through livestock trade or movement to new grazing fields) also occurs frequently in endemic regions in China [42], and this raises another concern for the current national control strategy.

### Diagnostic tests

While extensive control efforts in endemic areas have greatly suppressed the transmission levels, selective chemotherapy with praziquantel focusing on infected individuals becomes an important component of the national control program, and thus diagnosis of infection is a key step for determination of target populations for treatment, evaluation of morbidity, and assessment of control measures [43]. With decreasing infection intensities due to extensive control efforts, widely used direct parasitological techniques (e.g. miracidium hatch test (MHT) and Kato-Katz thick smear (KK)) have become increasingly insensitive [43]. KK and MHT are the two widely field-applied direct parasitological methods in China. The sensitivity of KK method ranges generally from 40% to 70%, while for MHT it varies from 24% to 95%, depending primarily on the levels of infection intensities [43]. The sensitivities of these direct parasitological techniques decrease generally with decrease in infection levels of humans. The insensitivities of KK and MHT methods lead to missing diagnosis of infected cases in areas of low endemicity, and in post-treatment situations. Other widely field-applied diagnostic alternatives are indirect immunodiagnostic assays (i.e. detecting schistosome-specific antibodies), e.g. Circumoval precipitin test (COPT), Indirect hemagglutination assay (IHA), Enzyme-linked immunosorbent assay (ELISA) and Dipstick dye immunoassay (DDIA). These antibody detection assays have relatively high sensitivities, but generally low specificities. The specificity varies widely for each test - 55% to 96% for COPT method, 35% to 94% for IHA method, 20% to 93% for ELISA method, and 33% to 97% for DDIA [43]. These antibody detection techniques cannot differentiate between current and past infections. Thus, the insensitivity or non-specificity of the currently field-applied diagnostic methods may result in difficulties in identifying true infected individuals for selective chemotherapy and assessing the effectiveness of interventions, collectively posing a significant obstacle in moving towards complete control of schistosomiasis. There are many other diagnostic methods such as clinical or ultrasonographic methods measuring the morbidity associated Schistosomiasis japonica, questionnaires, direct immunological tests detecting the schistosome-derived antigens, and the histological methods detecting schistosome eggs in tissue biopsies, but clinical or ultrasonographic methods, and questionnaires lack specificity in some areas such as China [43]. Although the circulating antigen detection assays have a relatively high specificity, they are not any better than the egg detection assays in terms of sensitivity in areas of low endemicity [44-47]. The histological diagnostic method with both high sensitivity and high specificity is neither simple nor convenient for population-based investigations. Recently, polymerase chain reaction (PCR) based techniques for detection of schistosome DNA in stool or sera and plasma have been developed, showing promise as a highly sensitive and specific assay for diagnosis of schistosome infection [48-55].

### Other neglected factors

Other neglected factors (e.g. praziquantel treatment failures, frequent flooding, and environmental and socio-economic changes) may also pose challenges to the current control program. The praziquantel treatment failures include treatment non-compliance and drug resistance. The chemotherapy with praziquantel has been implemented widely in endemic regions in China since the 1990s, especially during the 10-year World Bank Loan Project (WBLP) [56]. The compliance rates of chemotherapy drop in many communities after repeated rounds of mass or selective treatment [57]. Although currently there is no evidence of praziquantel resistance for *S. japonicum*, the resistance is found in other schistosoma species (e.g. *S. mansoni*) [56]. Frequent flooding along the Yangtze River and in the lake regions (e.g. Dongting Lake and Poyang Lake regions) have contributed to the spread of the intermediate host *Oncomelania hupensis*, and might counteract control efforts [4]. Environmental changes, caused by returning reclaimed land to the lake, construction and operation of Three Gorges dam, and the South-to-North water transfer project, might create new habitats for *O. hupensis*[4,58]*.*

### Discussion

Although bovines are largely responsible for transmission of *S. japonicum* in the lake and marshland regions of China*,* they are a major source of incomes for local farmers. The large marshland areas of Dongting Lake and Poyang Lake are a natural and ideal pasture for bovines and goats, and provide natural habitats for the intermediate snail host. It is difficult to remove all bovines from or completely fence pastures on the large marshland areas, since the farmers have herded bovines, especially water buffaloes, in the marshland areas for generations [17]. Even if all bovines were removed from or well fenced from the large marshland areas some time, it is also challenging to maintain the state of “without bovines” on the marshland areas for a long period of time because bovine movement also occurs frequently in the lakes and marshland regions due to livestock trade or new grazing fields [59]. So, fencing pastures or removing bovines often conflicts with the traditional way of raising livestock (especially water buffalo) and farmers’ incomes, and this considerable conflict might reduce the control efforts of the ongoing integrated control strategy, as fencing pastures is one of the key technical measures of the control strategy [3]. Hence, this integrated control strategy should be implemented in the light of local conditions of endemic areas for mutual benefit and development. For example, the environmental management should be encouraged to be implemented in some snail-inhabited marshland areas suitable for agricultural or forestry practices (e.g., Cropping cole or wheat, planting trees for controlling snails), thus, the farmers not only might increase their incomes, but also bovines could be well fenced by these modified snail-inhabited marshland areas, and snails could be controlled by the environmental management.

Although much is known about migration in China, the typology of population movement is complex and remains less well characterized [40]. There are at least two types of population movements related to schistosomiasis transmission in China (Table 2) [29-37]: a) fishermen, boatmen and herders who move around in rivers or lakes; b) People moving seasonally for paid work (e.g. 1) people from endemic areas moving into other endemic regions; 2) people from non-endemic areas moving to endemic areas; 3) from endemic areas to controlled areas; 4) from endemic areas to non-endemic areas but back to endemic areas). Tens of thousands of fishermen, boatmen and herders, with a high prevalence and intensity of *S. japonicum* infection, move around in Dongting Lake and Poyang Lake which are strongholds of schistosomiasis endemic areas in China (Table 2) [33,36,37]. These people frequently contact infected water, and their stools generally go into the water or snail habitats directly. It has been well-known that these migrant fishermen and bovines are responsible for a large proportion of the total contamination of the environment with *Schistosoma* eggs and are hence drivers in maintaining transmission of the disease in the lake and marshland areas of southern China [33]. The administration of chemotherapy or stool collection for these nomads is very difficult and little is known about the optimal treatment window for these nomads. Seasonal mobility for paid work has been very common in recent years in China, e.g. some rural young labourers from endemic areas leave their homes in the slack season (in farming), and return in the busy season (in farming), and other migrants only return to their home annually in the Chinese New Year. Some labourers from non-endemic regions enter endemic areas for agriculture in the busy season and leave in the slack season. In the current national control program of schistosomiasis, praziquantel-based chemotherapy is implemented generally only in endemic areas in autumn each year (i.e. slack season for farming), and the target population of chemotherapy is usually the local residents, not non-local people [3,14]. So, most of migrants infected with *S. japonicum* are missed for the treatment, and continue to be the infection sources. Hence, the target population and time of chemotherapy in the national control program of schistosomiasis should be adjusted in light of the patterns of population movement, for example, the target population of chemotherapy should include the non-local people infected with *S. japonicum*, and chemotherapy should also be implemented in the Chinese New Year when most of the infected migrants return to their home for the Spring Festival.

It is unclear whether schistosomiasis transmission could be maintained by these neglected factors in the absence of bovines. To address this question further study is needed, and the long-term effectiveness of the comprehensive national strategy should be also further evaluated [9]. For example, some longitudinal studies should be carried out to determine or evaluate whether these neglected factors are important for *S. japonicum* transmission.

## Conclusion

The current integrated strategy in China assumed that blocking the transmission of *S. japonicum* from human and cattle to snails would stem the transmission of this parasite [13]. Yet, the process of *S. japonicum* transmission is, in fact, relatively complicated, and might be abetted by other domesticated (e.g. dogs, cats, and goats) and wild (e.g. rats, and rabbits) animals, by population movement, and by social and hydrologic (e.g. flooding) links among some focal ‘hot spots’ of transmission. While we are working towards the transmission elimination of *S. japonicum*, these factors, which are currently neglected in the integrated strategy, need to be paid some attention by public health authorities.

## Competing interests

The authors declare that they have no competing interests.

## Authors’ contributions

YBZ, SL and QWJ wrote the manuscript together, YBZ summarized the Tables and all authors have read and approved the final version of the manuscript.

## References

[B1] Gryseels B Polman K Clerinx J Kestens L Human schistosomiasis Lancet 2006 368 1106 1118 10.1016/S0140-6736(06)69440-3 16997665

[B2] Gray DJ McManus DP Li YS Williams GM Bergquist R Ross AG Schistosomiasis elimination: lessons from the past guide the future Lancet Infect Dis 2010 10 733 736 10.1016/S1473-3099(10)70099-2 20705513

[B3] Wang LD Guo JG Wu XH Chen HG Wang TP Zhu SP Zhang ZH Steinmann P Yang GJ Wang SP Wu ZD Wang LY Hao Y Bergquist R Utzinger J Zhou XN China's new strategy to block Schistosoma japonicum transmission: experiences and impact beyond schistosomiasis Trop Med Int Health 2009 14 1475 1483 10.1111/j.1365-3156.2009.02403.x 19793080

[B4] Wang LD Utzinger J Zhou XN Schistosomiasis control: experiences and lessons from China Lancet 2008 372 1793 1795 10.1016/S0140-6736(08)61358-6 18930529 PMC7135384

[B5] Lin DD Hu GH Zhang SJ Optimal combined approaches of field intervention for schistosomiasis control in China Acta Trop 2005 96 242 247 10.1016/j.actatropica.2005.07.018 16125658

[B6] Jiang QW Wang LY Guo JG Chen MG Zhou XN Engels D Morbidity control of schistosomiasis in China Acta Trop 2002 82 115 125 10.1016/S0001-706X(02)00006-2 12020884

[B7] Yuan HC Jiang QW Zhao GM He N Achievements of schistosomiasis control in China Men Inst Oswaldo Cruz 2002 97 Suppl 1 187 189 10.1590/s0074-02762002000900036 12426618

[B8] Zhou XN Wang LY Chen MG Wu XH Jiang QW Chen XY Zheng J Utzinger J The public health significance and control of schistosomiasis - then and now Acta Trop 2005 2005 96 97 105 16125655 10.1016/j.actatropica.2005.07.005

[B9] Gray DJ Williams GM Li Y McManus DP Transmission dynamics of Schistosoma japonicum in the lakes and marshlands of China PLoS One 2008 3 e4058 10.1371/journal.pone.0004058 19115007 PMC2605259

[B10] Dai ZJ Yan JB Chen DR Xie ZM Xie DW Liao TB Study on epidemic factors and regularity of schistosomiasis japonica in mountainous district of liangshan yi autouomous prefecture Southest Chin J Agri Sci 1991 4 115 120 in Chinese

[B11] Zheng J Qian K Yao BY Zhu HQ Chen SH Zhang R Studies on distribution characteristics of infection sources of schistosomiasis in mountainous regions Chin J Schisto Control 1990 2 24 27 in Chinese

[B12] Davis GM Wu PW Chen HG Liu HY Guo JG Lin DD Lu SB Williams G Sleigh A Feng Z McManus DP A baseline study of importance of bovines for human Schistosoma japonicum infections around Poyang Lake, China: villages studied and snail sampling strategy Am J Trop Med Hyg 2002 66 359 371 12164289 10.4269/ajtmh.2002.66.359

[B13] Wang LD Chen HG Guo JG Zeng XJ Hong XL Xiong JJ Wu XH Wang XH Wang LY Xia G Hao Y Chin DP Zhou XN A strategy to control transmission of Schistosoma japonicum in China N Engl J Med 2009 360 121 128 10.1056/NEJMoa0800135 19129526

[B14] Zhou YB Liang S Chen GX Rea C He ZG Zhang ZJ Wei JG Zhao GM Jiang QW An integrated strategy for transmission control of Schistosoma japonicum in a marshland area of China: findings from a five-year longitudinal survey and mathematical modeling Am J Trop Med Hyg 2011 85 83 88 10.4269/ajtmh.2011.10-0574 21734130 PMC3122349

[B15] Seto EYW Remais JV Carlton EJ Wang S Liang S Brindley PJ Qiu D Spear RC Wang LD Wang TP Chen HG Dong XQ Wang LY Hao Y Bergquist R Zhou XN Toward sustainable and comprehensive control of schistosomiasis in China: lessons from Sichuan PLoS Negl Trop Dis 2011 5 e1372 10.1371/journal.pntd.0001372 22039563 PMC3201916

[B16] Lu DB Wang TP Rudge JW Donnelly CA Fang GR Webster JP Contrasting reservoirs for Schistosoma japonicum between marshland and hilly regions in Anhui, China–a two-year longitudinal parasitological survey Parasitology 2010 137 99 110 10.1017/S003118200999103X 19723358

[B17] He HB Thought of schistosomiasis control strategy with emphasis on controlling sources of infection in lake and marshland endemic regions Chin J Schisto Control 2011 23 710 713 22379836

[B18] Wang TP Shrivastava J Johansen MV Zhang SQ Wang FF Webster JP Does multiple hosts mean multiple parasites? Population genetic structure of Schistosoma japonicum between definitive host species Int J Parasit 2006 2006 36 1317 1325 10.1016/j.ijpara.2006.06.011 16876170

[B19] Xu GY Tian JC Chen GM Yang HM Qiu L Hu HB Observation on natural focal disease of schistosomiasis in rattus norvegicus in Nanjing J Pract Parasit Dis 1999 7 4 6 in Chinese

[B20] Wang TP Guo JH Wu WC Zhang SQ Lu DB Zhang GH The infection sources of schistosomiasis japonica and their role in the transmission of this disease in marshland areas, Anhui province Parasit Prev Res 1997 26 138 140 in Chinese

[B21] Sun LP Hong QB Cao Q Gu BL Gao ZH Guo BY Role of different infection sources in the transmission of schistosomiasis japonica in marshland region Chin J Schisto Control 1997 9 44 45 in Chinese

[B22] Xu FS Gu XG Zhao WX Li YX Yin HZ Zhao LG Role of different infection sources in the transmission of schistosomiasis japonica in mountainous region J Pract Parasit Dis 1995 3 129 in Chinese

[B23] Su ZW Hu CQ Fu Y Chen W Huan XB Role of several host in transmission of schistosomiasis japonica in lake region Chin J Parasitol Parasit Dis 1994 12 48 51 in Chinese

[B24] Gu XG Zhao WX Xu FS Qiu DC Han Y Chen GY Analysis on epidemiology of schisotosomiasis in mountainous terrace regions in Sichuan through a typical investigation Chin J Schisto Control 1993 5 82 84 in Chinese

[B25] Chen DJ Gong ZB Epidemiological survey of schistosomiasis in high mountainous region in Eryuan county of Yunnan province Chin J Schisto Control 1989 1 10 13 in Chinese

[B26] Yao BY Zheng J Qian K Chen SH Zhu HQ Wu WP The role of the animal host in the epidemiology of schistosomiasis in mountainous regions Chin J Schisto Control 1989 1 1 3 in Chinese

[B27] Riley S Carabin H BeLisle P Joseph L Tallo V Balolong E Willingham AL Fernandez TJ Gonzales RO Olveda R McGarvey ST Multi-host transmission dynamics of Schistosoma japonicum in Samar Province, the Philippines PLoS Med 2008 5 e18 10.1371/journal.pmed.0050018 18215106 PMC2211559

[B28] Rudge JW Carabin H Balolong E Tallo V Shrivastava J Lu DB Basáñez MG Olveda R McGarvey ST Webster JP Population genetics of schistosoma japonicum within the Philippines suggest high levels of transmission between humans and dogs PLoS Negl Trop Dis 2008 2 e340 10.1371/journal.pntd.0000340 19030225 PMC2582952

[B29] Chen GX He ZG Han SM Investigation on prevalent trends and analysis of control period among floating population in schistosomiasis endemic areas of Guichi District Chin J Schisto Contro 2011 23 148 153 in Chinese 22164613

[B30] Wang ZC Sun WS Xiong YQ Tian JP Zhang J Zhang W Sero-epidemiological survey on floating population immigrated from schistosomiasis free areas to Hyper-endemic marshland area Parasit Infect Dis 2008 6 127 130 in Chinese

[B31] He JC Zhang SQ Wang TP Chen GX Cui DY He ZG Investigation on schistosome infection among migrant workers from rural areas Chin J Schisto Control 2008 20 114 116 in Chinese

[B32] Zhang YQ Zhang R Qi LJ Zhang J Shu RR He QX Survey on schistosomiasis among farmers working outside native farmland in rural area of Qianjiang city Parasit Infect Dis 2003 1 62 64 in Chinese

[B33] Li YS He YK Zeng QR McManus DP Epidemiological and morbidity assessment of Schistosoma japonicum infection in a migrant fisherman community, the Dongting Lake region, China Trans R Soc Trop Med Hyg 2003 97 177 181 10.1016/S0035-9203(03)90112-X 14584373

[B34] Chen GX Wang MS Han SM Investigation of movement population infected with schistosome in the endemic areas of Guichi city Chin J Schisto Control 2001 13 102 103 in Chinese

[B35] Zhang SQ Li QY Wang TP Zhang GH Wu WD Ge JH Epidemiological survey of movement population infected with schistosomiasis in marshland area Chin J Zoon 1998 14 85 86 in Chinese

[B36] Zheng J Guo JG Zhu HQ Movement population and schistosomiasis transmission Chin J Schisto Control 1999 11 125 127 in Chinese

[B37] Ross AG Yuesheng L Sleigh AS Yi L Williams GM Wu WZ Wu WZ Xinsong L Yongkang H McManus DP Epidemiologic features of Schistosoma japonicum among fishermen and other occupational groups in the Dongting Lake region (Hunan Province) of China Am J Trop Med Hyg 1997 57 302 308 9311640 10.4269/ajtmh.1997.57.302

[B38] Alirol E Getaz L Stoll B Chappuis F Loutan L Urbanisation and infectious diseases in a globalised world Lancet Infect Dis 2011 11 131 141 10.1016/S1473-3099(10)70223-1 21272793 PMC7106397

[B39] Aagaard-Hansen J Nombela N Alvar J Population movement: a key factor in the epidemiology of neglected tropical diseases Trop Med Int Health 2010 15 1281 1288 10.1111/j.1365-3156.2010.02629.x 20976871

[B40] Ren FF Fu HP Liu M Liang WN The classification of population mobility and impact on infectious diseases Soft Science of Health 2010 24 272 276 in Chinese

[B41] Cao CL Guo JG Schistosome infection and control of migrant population Chin J Schisto Control 2010 22 388 390 in Chinese

[B42] Kloos H Correa-Oliveira R dos Reis DC Rodrigues EW Monteiro LA Gazzinelli A The role of population movement in the epidemiology and control of schistosomiasis in Brazil: a preliminary typology of population movement Mem Inst Oswaldo Cruz 2010 105 578 586 10.1590/S0074-02762010000400038 20721511

[B43] Zhou YB Zheng HM Jiang QW A diagnostic challenge for schistosomiasis japonica in China: consequences on praziquental-based morbidity control Parasit Vectors 2011 4 194 10.1186/1756-3305-4-194 21981948 PMC3195757

[B44] Guan X Shi Y Collaborative study on evaluation of immunodiagnostic assays in Schistosomiasis japonica by treatment efficacy assessment Collaboration Group Chin Med J 1996 109 659 664 9275331

[B45] Attallah AM Ismail H El Masry SA Rizk H Handousa A El Bendary M Tabll A Ezzat F Rapid detection of a Schistosoma mansoni circulating antigen excreted in urine of infected individualsby using a monoclonal antibody J Clin Microbiol 1999 37 354 357 9889217 10.1128/jcm.37.2.354-357.1999 PMC84306

[B46] De Jonge N Gryseels B Hilberath GW Polderman AM Deelde AM Detection of circulating anodic antigen by ELISA for seroepidemiology of schistosomiasis mansoni Trans R Soc Trop Med Hyg 1988 82 591 594 10.1016/0035-9203(88)90523-8 3151417

[B47] Van Lieshout L Panday UG de Jonge N Krijger FW Oostburg BF Polderman AM Deelder AM Immunodiagnosis of schistosomiasis mansoni in a low endemic area in Surinam by determination of the circulating antigens CAA and CCA Acta Trop 1995 59 9 29 10.1016/0001-706x(94)00084-e 7785523

[B48] Ibironke OA Phillips AE Garba A Lamine SM Shiff C Diagnosis of Schistosoma haematobium by detection of specific DNA fragments from filtered urine samples Am J Trop Med Hyg 2011 84 998 1001 10.4269/ajtmh.2011.10-0691 21633040 PMC3110375

[B49] Oliveira LM Santos HL Gonçalves MM Barreto MG Peralta JM Evaluation of polymerase chain reaction as an additional tool for the diagnosis of low-intensity Schistosoma mansoni infection Diagn Microbiol Infect Dis 2010 68 416 421 10.1016/j.diagmicrobio.2010.07.016 20884153

[B50] Gomes LI Dos Santos Marques LH Enk MJ de Oliveira MC Coelho PM Rabello A Development and evaluation of a sensitive PCR-ELISA system for detection of schistosoma infection in feces PLoS Negl Trop Dis 2010 4 e664 10.1371/journal.pntd.0000664 20421918 PMC2857640

[B51] Xu J Rong R Zhang HQ Shi CJ Zhu XQ Xia CM Sensitive and rapid detection of Schistosoma japonicum DNA by loop-mediated isothermal amplification (LAMP) Int J Parasitol 2010 40 327 331 10.1016/j.ijpara.2009.08.010 19735662

[B52] Allam AF Kader O Zaki A Shehab AY Farag HF Assessing the marginal error in diagnosis and cure of Schistosoma mansoni in areas of low endemicity using Percoll and PCR techniques Trop Med Int Health 2009 14 316 321 10.1111/j.1365-3156.2009.02225.x 19278527

[B53] Wichmann D Panning M Quack T Kramme S Burchard GD Grevelding C Drosten C Diagnosing schistosomiasis by detection of cell-free parasite DNA in human plasma PLoS Negl Trop Dis 2009 3 e422 10.1371/journal.pntd.0000422 19381285 PMC2667260

[B54] Sandoval N Siles-Lucas M Pérez-Arellano JL Carranza C Puente S López-Abán J Muro A A new PCR-based approach for the specific amplification of DNA from different Schistosoma species applicable to human urine samples Parasitology 2006 133 581 587 10.1017/S0031182006000898 16834820

[B55] Pontes LA Dias-Neto E Rabello A Detection by polymerase chain reaction of Schistosoma mansoni DNA in human serum and feces Am J Trop Med Hyg 2002 66 157 162 12135287 10.4269/ajtmh.2002.66.157

[B56] Wang W Dai JR Li HJ Shen XH Liang YS Is there reduced susceptibility to praziquantel in Schistosoma japonicum? Evidence from China Parasitology 2010 137 1905 1912 10.1017/S0031182010001204 20810006

[B57] Guo JG Hu GH Xiong YL Compliance analysis of the residents with mass medical treatment in areas highly endemic for schistosomiasis Chin J Parasitol Parasit Dis 2000 18 58 59 in Chinese 12567481

[B58] Liang YS Wang W Li HJ Shen XH Xu YL Dai JR The South-to-North Water Diversion Project: effect of the water diversion pattern on transmission of Oncomelania hupensis, the intermediate host of Schistosoma japonicum in China Parasit Vectors 2012 5 52 10.1186/1756-3305-5-52 22433070 PMC3325841

[B59] Zheng J Guo JG Wang XF Zhu HQ Relationship of the livestock trade to schistosomiasis transmission in mountainous area Chin J Parasitol Parasit Dis 2000 18 146 148 in Chinese 12567688

